# Functional and Neuropathological Evidence for a Role of the Brainstem in Autism

**DOI:** 10.3389/fnint.2021.748977

**Published:** 2021-10-20

**Authors:** Joan S. Baizer

**Affiliations:** Department of Physiology and Biophysics, Jacobs School of Medicine and Biomedical Sciences, University at Buffalo, Buffalo, NY, United States

**Keywords:** inferior olive, arcuate nucleus of the medulla, pontine nuclei, cerebellum, vestibular nuclear complex, cochlear nuclear complex

## Abstract

The brainstem includes many nuclei and fiber tracts that mediate a wide range of functions. Data from two parallel approaches to the study of autistic spectrum disorder (ASD) implicate many brainstem structures. The first approach is to identify the functions affected in ASD and then trace the neural systems mediating those functions. While not included as core symptoms, three areas of function are frequently impaired in ASD: (1) Motor control both of the limbs and body and the control of eye movements; (2) Sensory information processing in vestibular and auditory systems; (3) Control of affect. There are critical brainstem nuclei mediating each of those functions. There are many nuclei critical for eye movement control including the superior colliculus. Vestibular information is first processed in the four nuclei of the vestibular nuclear complex. Auditory information is relayed to the dorsal and ventral cochlear nuclei and subsequently processed in multiple other brainstem nuclei. Critical structures in affect regulation are the brainstem sources of serotonin and norepinephrine, the raphe nuclei and the locus ceruleus. The second approach is the analysis of abnormalities from direct study of ASD brains. The structure most commonly identified as abnormal in neuropathological studies is the cerebellum. It is classically a major component of the motor system, critical for coordination. It has also been implicated in cognitive and language functions, among the core symptoms of ASD. This structure works very closely with the cerebral cortex; the cortex and the cerebellum show parallel enlargement over evolution. The cerebellum receives input from cortex via relays in the pontine nuclei. In addition, climbing fiber input to cerebellum comes from the inferior olive of the medulla. Mossy fiber input comes from the arcuate nucleus of the medulla as well as the pontine nuclei. The cerebellum projects to several brainstem nuclei including the vestibular nuclear complex and the red nucleus. There are thus multiple brainstem nuclei distributed at all levels of the brainstem, medulla, pons, and midbrain, that participate in functions affected in ASD. There is direct evidence that the cerebellum may be abnormal in ASD. The evidence strongly indicates that analysis of these structures could add to our understanding of the neural basis of ASD.

## Introduction

The goal of this review is to consider a possible role of the brainstem in autism or autistic spectrum disorder (ASD). The question of brainstem involvement is complex; the “brainstem” includes many structures and fiber tracts mediating a wide range of functions including sensory, motor, and affective. To develop hypotheses about the possible involvement of brainstem structures in ASD, we will first consider the implications of data from two complementary experimental approaches. The first approach has been to describe the functions are impaired in ASD; the next step then is to look at the neural systems mediating those functions, focusing on the brainstem components. The second approach has been to directly identify brain structures that affected in ASD brains, the next step from those data is to consider the afferent and efferent connections of those structures, again with a focus on brainstem relays. The perspective here is on the participation of the brainstem in circuitry in the adult brain. Another, complementary, perspective is discussed extensively by Dadalko and Travers (2018) who consider the role of brainstem structures in the development of the brain.

### The First Question Is What Is ASD?

ASD is a neurodevelopmental disorder, thought to reflect abnormal brain development (DiCicco-Bloom et al., 2006; Geschwind, 2011). Symptoms are not usually apparent at birth, but emerge by about the age of 2–3 years old (Filipek et al., 1999; DiCicco-Bloom et al., 2006). There are some studies that argue for subtle earlier manifestations (Zwaigenbaum et al., 2005, 2013). ASD is a complicated diagnosis with much individual variability (this has been discussed by many authors, some examples: Ciaranello and Ciaranello, 1995; Filipek et al., 1999; DiCicco-Bloom et al., 2006; Grzadzinski et al., 2013; Zwaigenbaum et al., 2013; Lai et al., 2014; CDC, 2021). The diagnosis is based on behavioral analysis and not on genetics or biomarkers (discussion in Geschwind, 2011). At present, the causes of ASD are not understood; there is clearly a genetic component, but the genetics are complex (Folstein and Rutter, 1977a,b; Folstein and Rosen-Sheidley, 2001; Geschwind, 2011; Huguet et al., 2016). Patients with known genetic syndromes can meet the criteria for an ASD diagnosis, these syndromes include Down Syndrome, (DS; trisomy 21, Hepburn et al., 2008), Fragile X syndrome (FXS; Hoeft et al., 2011), Timothy Syndrome (Bett et al., 2013), Tuberous Sclerosis (Smalley, 1998), and Rett syndrome (Percy, 2011). In addition, there are many other genes with mutations associated with a risk of ASD (Campbell et al., 2007; Geschwind, 2011; Jiang et al., 2013; Pinto et al., 2014; Myers et al., 2020; Chawner et al., 2021). Data from twin studies suggest that there are non-genetic in addition to genetic factors determining the emergence of ASD (Kates et al., 2004). ASD is also associated with other neurological conditions like seizure disorders (about 39%) and intellectual disability (ID, about 45% Wegiel et al., 2012). Finally, ASD is a sexually dimorphic disorder, affecting more males than females (about 4:1, Folstein and Rosen-Sheidley, 2001; Yamasue et al., 2009), raising the possibility of sexually dimorphic effects in the brain. Thus the ASD population is medically, behaviorally, and genetically very heterogeneous.

### Functions Affected in ASD

The criteria for the diagnosis of autism have changed over the years. The most recent criteria for Autism Spectrum Disorder are deficits in two areas (1) Social communication and interaction across multiple contexts and (2) Restricted and repetitive behaviors (DSM-5, 2013; see also Lai et al., 2014). However, there are many additional functional deficits that have been described in subsets of ASD patients. We will focus on three aspects of function whose neural substrates include brainstem nuclei: (1) Motor control both of the limbs and body and of the eyes. (2) Auditory and vestibular information processing. (3) Control of affect.

#### Motor Symptoms in ASD: Limbs and Body

While not a core deficit of ASD, problems with different aspects of motor control have been reported in many studies. Symptoms described include abnormal development of motor milestones, difficulties in postural control and gait, toe-walking, difficulty in learning to ride a bicycle, poor motor coordination and “clumsiness,” dystonia or hypotonia, difficulties with motor learning, rigidity and repetitive and stereotyped motor behaviors like hand-flapping and rocking, and motor memory (Kohenraz et al., 1992; Ciaranello and Ciaranello, 1995; Teitelbaum et al., 1998; Dawson et al., 2000; Ming et al., 2007; Albinali et al., 2009; Boyd et al., 2012; MacDonald et al., 2012; Marko et al., 2015; Hannant et al., 2016; Eggleston et al., 2017; Bruchage et al., 2018; Bell et al., 2019; Neely et al., 2019; Bojanek et al., 2020). Motor symptoms are prevalent enough that some authors have proposed that motor deficits should be considered among the core symptoms of ASD (Mosconi and Sweeney, 2015). The severity of motor symptoms may correlate with impairments in cognitive and language domains (Bhat, 2021), suggesting that they reflect the overall atypical brain development. The wide range of motor symptoms again reflects the heterogeneity of ASD.

#### Motor Symptoms in ASD: Control of Eye Movements

There are many reports of eye movement abnormalities in ASD (reviews in Sweeney et al., 2004; Mosconi and Sweeney, 2015). Deficits have been reported in all types of voluntary eye movements: saccades, smooth pursuit and maintenance of fixation, but the exact nature of the deficits varies among studies. For saccadic eye movements, Rosenhall et al. (1988) tested saccades to targets and found hypometric saccades and reduced saccade velocity in ASD subjects. Takarae et al. (2004b) measured the accuracy of visually guided saccades and found greater variability in saccadic accuracy in ASD but no effects of saccade latency or velocity. Zalla et al. (2018) used a complex test of saccadic eye movement accuracy and found reduced saccadic gain and reduced peak saccade velocity in ASD. For smooth pursuit, Takarae et al. (2004a, 2008) found deficits in accurate tracking of moving targets and found longer latency and more “catch-up” saccades in ASD subjects. Deficits were also found in “saccadic adaptation” in a task in which the saccade target is moved before the target is acquired, a task eliciting learning in typical subjects (Mosconi et al., 2013). Nowinski et al. (2005) studied the ability to maintain fixation on visual targets and found more “intrusive saccades” in a task requiring subjects to maintain fixation on a “remembered” (no longer visible) target. Wegiel et al. (2013) reported poor or no eye contact in ASD subjects. Overall the data support the hypothesis that control of eye movements is affected in ASD.

#### Sensory Processing: Auditory and Vestibular Systems

Sensory processing deficits, specifically of auditory and vestibular information, are also characteristic of ASD (discussion and additional references in Baranek et al., 2006; Lane et al., 2010; Mansour and Kulesza, 2020). While peripheral hearing loss is not found (Beers et al., 2014), there are many studies showing auditory dysfunction in children with ASD (references in Rimland and Edelson, 1995; Lukose et al., 2013; Kozou et al., 2018; Smith et al., 2019). Lukose et al. (2013) studied the acoustic stapedial reflex (ASR: contraction of the stapedius muscle of the middle ear in response to loud sounds) and found lower thresholds and longer latencies in ASD subjects. A number of auditory training schemes have been proposed to improve auditory processing (Rimland and Edelson, 1995; Russo et al., 2010; Gopal et al., 2020).

Several observations also suggest differences in processing vestibular stimuli (Kern et al., 2007). Some ASD behaviors like rocking (Dawson et al., 2000; Albinali et al., 2009) would increase vestibular input. Vestibular input is also critical for several motor functions including postural stability, another function affected in ASD (Molloy et al., 2003; Bojanek et al., 2020). Vestibular therapy is often proposed for children with ASD (Smoot Reinert et al., 2015).

#### Regulation of Affect

Atypical regulation of affect and attention are well-documented ASD symptoms (Harris et al., 1999; Konstantareas and Stewart, 2006; Mazefsky et al., 2013; Mazefsky and White, 2014).

We will return to a consideration of the circuitry underlying these functions after looking at what is known about changes in the brain in ASD.

### The Brain in ASD

Many hypotheses about the neural basis of ASD postulate that the diverse symptoms reflect dysfunction in multiple (but, importantly, not all) brain regions and/or systems. Such dysfunction could have multiple manifestations: (1) Macroscopic structural differences in specific brain regions, including differences in axon tracts (numbers or diameters of axons). (2) Microscopic differences in neuronal structure. (3) Physiological differences affecting the action of critical circuits; such could arise from abnormalities in transmitters, receptors, and/or transporters. This perspective differentiates ASD from other genetically determined neurological diseases, e.g., Krabbe disease (Hunter’sHope, 2021) or FXS (Hagerman et al., 2017) where a single gene mutation affecting a single protein results in global effects on most or all neurons across multiple systems and structures.

There are many constraints in studying the human brain; invasive physiological and neuroanatomical (especially tract-tracing) techniques so extensively used in animal studies cannot be used in humans. The two major approaches for studying the human brain are (1) Postmortem examination of brains using histological techniques and (2) Analysis of brains in living subjects using imaging techniques.

#### Neuropathological Studies

Postmortem study of individual brains allows direct histological examination of the brain at the cellular/neurochemical level. The limitations of these studies are that only a few brains have been available for study, and the information about the range of ASD symptoms in any individual may be limited. The diversity of ASD symptoms and causes is mirrored by a diversity of neuropathological reports (summary in Palmen et al., 2004).

#### Imaging Studies in ASD

Imaging techniques include MRI to show structure, fMRI to show functional activation and DT-MRI to show connections. These studies can include relatively large numbers of ASD subjects and usually also include equivalent numbers of control subjects (review and references in Ecker, 2017; Dadalko and Travers, 2018).

##### Limitations: Subject selection

Integrating data from the many imaging, behavioral, and neuroanatomical studies of ASD is complicated by the fact that different studies use very different criteria for selection of subjects and of controls. Some studies include subjects with syndromes associated with ASD like DS or FXS (e.g., Kaufmann et al., 2003). Other studies explicitly exclude such subjects (for example Müller et al., 2001; Nowinski et al., 2005; Neely et al., 2019; Unruh et al., 2019). The neuroanatomical substrates for ASD in genetically different populations may be different. Hoeft et al. (2011) found differences between FXS and idiopathic ASD boys in the volume of cerebral gray matter and white matter in different cortical regions. Many behavioral and imaging studies are limited to subjects who are “high-functioning autistic”/Asperger’s Syndrome (examples include Nowinski et al., 2005; Langen et al., 2007; Takarae et al., 2007; Catani et al., 2008). Some include only people with normal IQs (e.g., Hua et al., 2013; Oldehinkel et al., 2019). “Lower-functioning” individuals may have had behavior incompatible with the demands of behavioral or imaging studies, and in fact some studies have used sedation for structural imaging of ASD subjects (Sparks et al., 2002; Carper and Courchesne, 2005; Schumann et al., 2009; Zielinski et al., 2014; Lange et al., 2015); the necessity for sedation could further limit subject selection. The problems with the selection of appropriate controls have been thoughtfully discussed by Jarrold and Brock (2004). Therefore, there may be a population of lower IQ/behaviorally challenging ASD individuals excluded from many studies, especially imaging studies with behavioral demands. Post-mortem brain analyses probably include a higher percentage of ASD with Intellectual Disability (ID, for example Bailey et al., 1998) and/or behavioral challenges than do imaging studies. These issues complicate the efforts of trying to understand the biological basis of ASD in light of the heterogeneity in ASD characteristics and correlates.

Another concern with subject selection is that many studies use only male subjects (some examples: Müller et al., 2001; Schumann et al., 2004; Catani et al., 2008; Zielinski et al., 2014; Igelstrom et al., 2017), potentially missing sexually dimorphic anomalies. That such may exist is suggested by Libero et al. (2016) who found differences in “disproportionate megalencephaly” between girls and boys, with the boys affected and the girls not. A related problem concerns the variability in criteria that have been used for establishing an ASD diagnosis; issues of diagnosis and subject selection are summarized by Simmons et al. (2009).

## What Do We Know About the Brain in ASD: Candidate Structures and Their Connections

### The Cerebellum in ASD

Many studies have found abnormalities of the cerebellum in ASD; data come from both neuropathological and imaging studies (Ritvo et al., 1986; Murakami et al., 1989; Bauman, 1991; Courchesne et al., 1994, 2011; Ciesielski et al., 1997; Bailey et al., 1998; Levitt et al., 1999; Purcell et al., 2001; Fatemi et al., 2002, 2012; Kaufmann et al., 2003; Allen et al., 2004; Palmen et al., 2004; Allen, 2005; Catani et al., 2008; Whitney et al., 2009; Yip et al., 2009; Wegiel et al., 2010, 2012, 2014; Donovan and Basson, 2017; Bruchage et al., 2018). However, different studies report different cerebellar abnormalities. The most common deficits seen in microscopic analysis of the cerebellum are loss of Purkinje cells and granule cells in cerebellar cortex, loss or abnormal appearance of neurons in the deep cerebellar nuclei and differences in the size of vermal lobules (Bauman, 1991; Kemper and Bauman, 2002; Whitney et al., 2009; Wegiel et al., 2014). Wegiel et al. (2013) reported dysplasia of the flocculus, a part of the cerebellum involved in eye movement control (Zee et al., 1981).

Structural imaging studies likewise have found differences in the size of parts of the vermis, but which lobules were affected and in which direction (bigger vs. smaller than in controls) varies among studies (Courchesne et al., 1994; Harris et al., 1999; Levitt et al., 1999; Kaufmann et al., 2003; Kates et al., 2004; Marko et al., 2015). Cerebellar hemispheres, as well as the vermis, may be smaller (Murakami et al., 1989). Catani et al. (2008) reported that the efferent pathway from the cerebellum, the superior cerebellar peduncle, is smaller in ASD. Hanaie et al. (2013) using DTI found structural differences in the superior cerebellar peduncles between ASD and control subjects; differences correlated with deficits in motor skills. Functional imaging data also confirm differences in cerebellar involvement between control and ASD subjects in a simple motor task (Allen et al., 2004).

### Connections of the Cerebellum: The Brainstem

There is thus strong and consistent evidence that the cerebellum is affected in ASD. What is the significance of this result? The classical role of the cerebellum is in motor control (Evarts and Thach, 1969), including the control of eye movements (Robinson and Fuchs, 2001; Blazquez and Pastor, 2013). More recently, a role of the cerebellum in language and cognition has been proposed on the basis of anatomical and clinical studies (Strick et al., 2009; Buckner, 2013). Cerebellar dysfunction in ASD could therefore contribute to cognitive/language as well as motor deficits (Allen, 2005).

**FIGURE 1:**
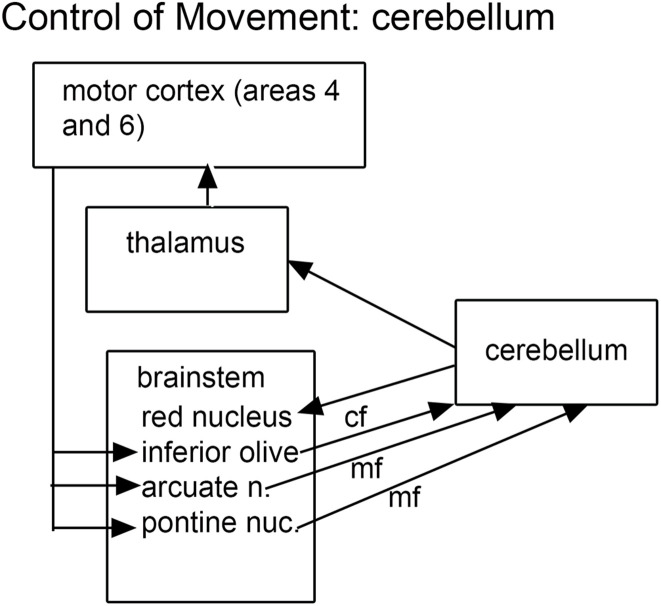
The brainstem inputs and outputs of the cerebellum. cf, climbing fibers; mf, mossy fibers.

The findings of cerebellar abnormalities in ASD dictate consideration of its connections, and these connections make a compelling argument for the role of the brainstem in ASD. The cerebellum is connected to the brain by relays in brainstem and diencephalon. The cerebellum receives information from several “precerebellar” brainstem relays; the output of the cerebellum is via the neurons of the cerebellar deep nuclei that project to multiple brainstem structures (Asanuma et al., 1983a). Thus the neuroanatomical data suggest that both precerebellar brainstem structures as well as the brainstem targets of cerebellar outflow might be affected in ASD brains. What are these structures? Figure 1 summarizes the critical connections of the cerebellum.

### Precerebellar Brainstem Structures: Inferior Olive, Pontine Nuclei, and the Arcuate Nucleus

**FIGURE 2:**
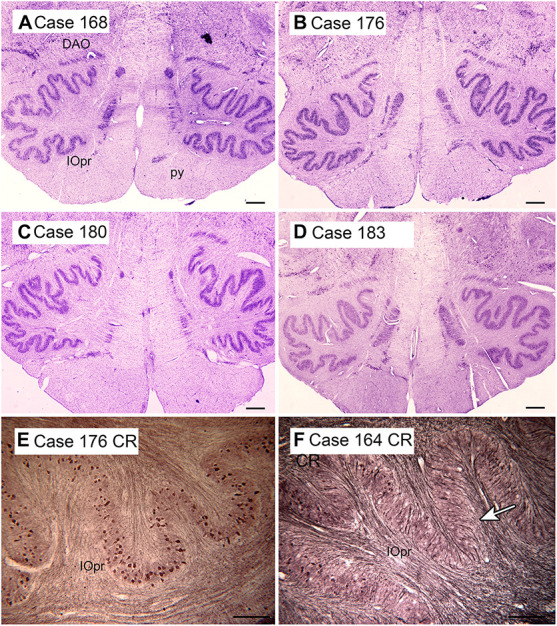
Variability in the shape of the IOpr in humans. **(A–D)** The IOpr in cresyl violet stained transverse sections of the brainstem in four cases. Note the differences in the folding pattern among cases and the left-right asymmetry in each case. Scale bars = 1 mm. **(E,F)** Variability in the density of neurons expressing the calcium-binding protein calretinin (CR) illustrated in two cases. The arrow in **(F)** shows a region with few immunostained neurons. Scale bars = 250 μm. CR, calretinin; DAO, dorsal accessory olive; IOpr, principal nucleus of the inferior olive. Figures 2, 3, 6, and 8 show photomicrographs of sections that were prepared for several different projects on the human brainstem. Details of the methods are given in earlier publications (Baizer and Broussard, 2010; Baizer et al., 2011a,b, 2014, 2018a,b, 2021).

The cerebellum receives two kinds of afferent fibers, mossy fibers and climbing fibers (Eccles, 1967). The inferior olive is the sole source of climbing fibers that innervate the cerebellum and is also the recipient of feedback projections from the cerebellum. There is evidence for IO abnormalities in ASD in a few neuropathological cases (Bailey et al., 1998; Kemper and Bauman, 2002). However, interpretation of the results for the IO is complex. We found individual variability in the appearance of neurons and in the expression of calcium-binding proteins in normal subjects (Baizer et al., 2011b, 2018b). The age-pigment lipofuscin is especially prominent in IO neurons, and may correlate with age-related degenerative changes in IO neurons affecting protein expression (Mann and Yates, 1974; Mann et al., 1978). Figure 2 illustrates the variability in shape and neurochemical properties of the IOpr in humans.

**FIGURE 3:**
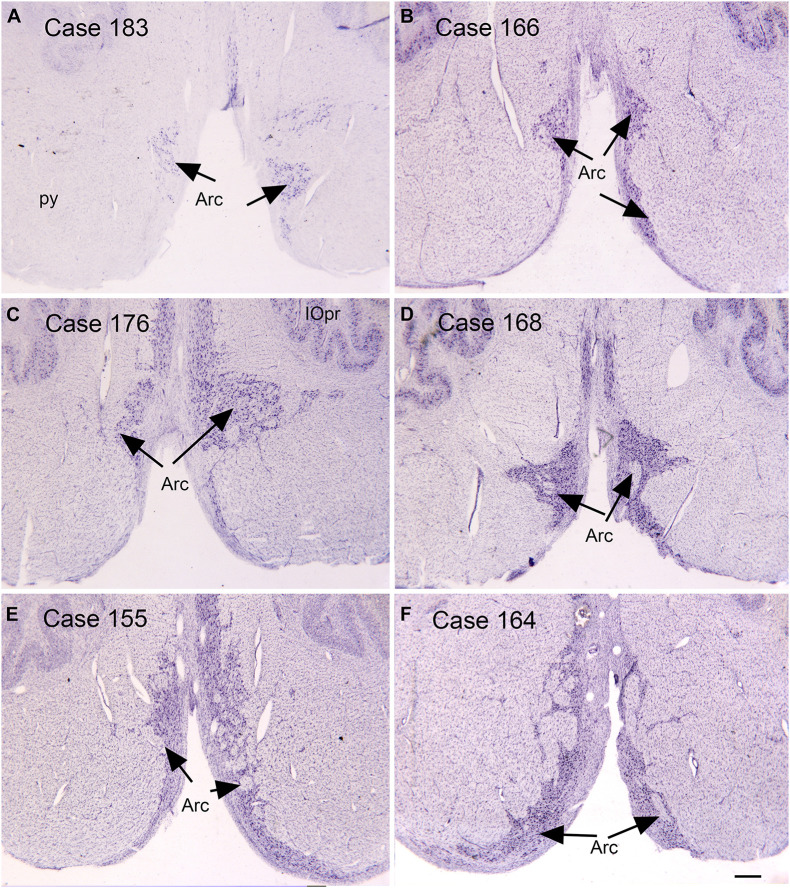
Variability in the arcuate nucleus. **(A–F)** The arcuate nucleus (Arc, arrows) on cresyl violet stained transverse sections of the human brainstem. Note the differences in size and shape among cases, and the left-right asymmetry within cases. Scale bar = 0.5 mm. Arc, arcuate nucleus; IOpr, principal nucleus of the inferior olive; py, pyramidal tract.

**FIGURE 4:**
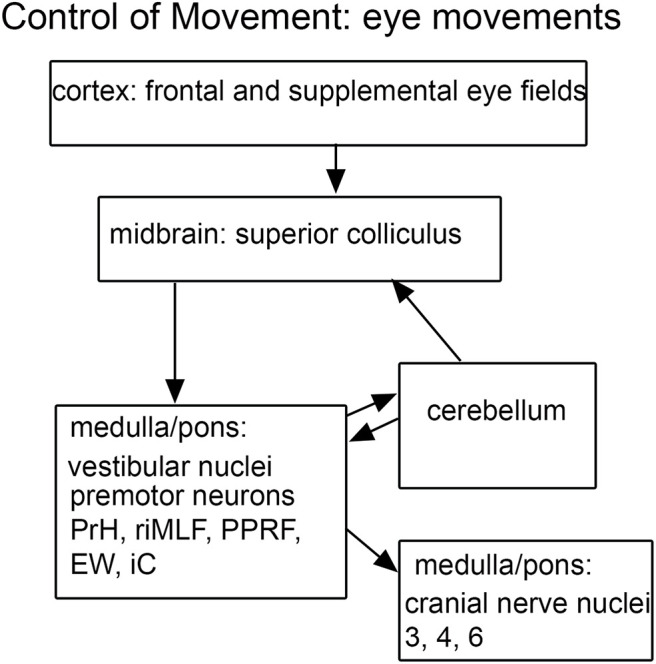
Schematic of brainstem nuclei critical for eye movement control. Cranial nerve nuclei 3-oculomotor, 4-trochlear, 6-abducens; EW, Edinger-Westphal nucleus; iC, interstitial nucleus of Cajal; PrH, nucleus prepositus hypoglossi; PPRF, paramedian pontine reticular formation; riMLF, rostral interstitial nucleus of the medial longitudinal fasciculus; SNr, substantia nigra, pars reticulata.

**FIGURE 5:**
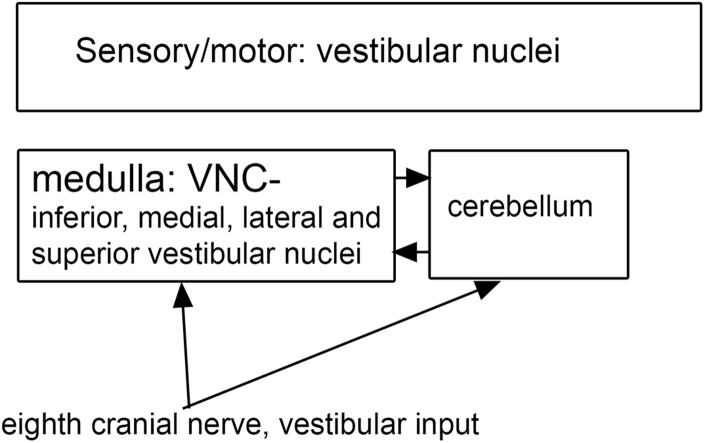
Vestibular input to the vestibular nuclei of the brainstem.

**FIGURE 6:**
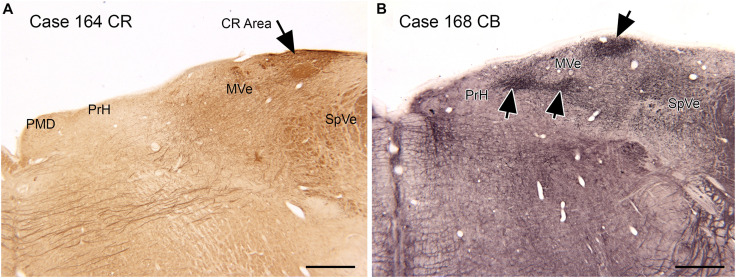
Compartments in the medial vestibular nucleus (MVe) marked by immunoreactivity to calretinin (CR, **A**, arrow) and calbindin (CB, **B**, arrows). Scale bars = 1 mm. PrH, nucleus prepositus hypoglossi; PMD, nucleus paramedianus dorsalis; SpVe, spinal or inferior vestibular nucleus.

**FIGURE 7:**
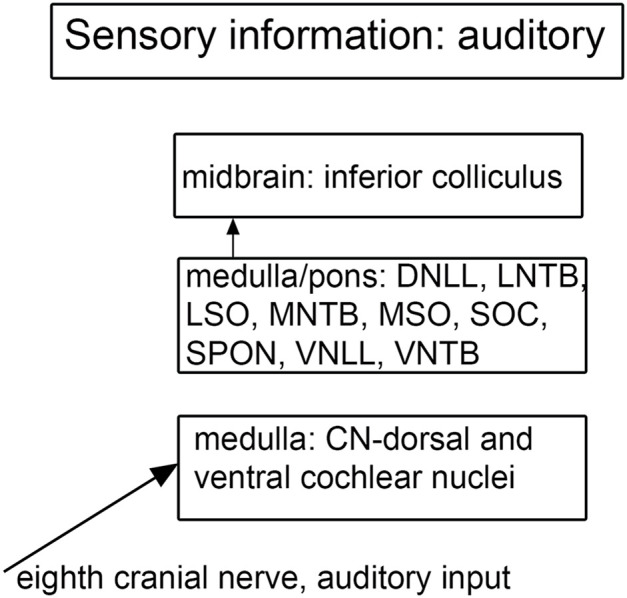
Schematic showing the main brainstem nuclei of the auditory system. CN, cochlear nuclei; DNLL, dorsal nucleus of the lateral lemniscus; MNTB, medial nucleus of the trapezoid body; MSO, medial superior olive; SOC, superior olivary complex; SPON, superior paraolivary nucleus; VNLL ventral nucleus of the lateral lemniscus; VNTB, ventral nucleus of the trapezoid body.

**FIGURE 8:**
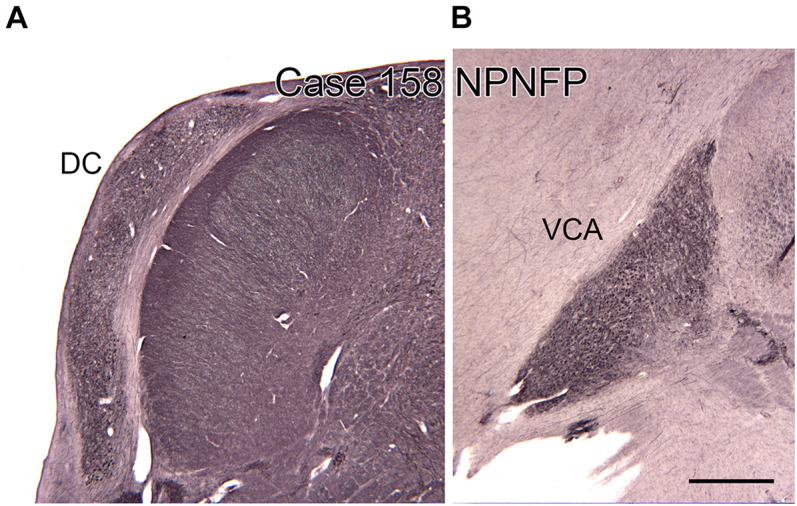
The dorsal (DC, **A**) and ventral (VCA, **B**) cochlear nuclei in the human brain shown on transverse sections immunostained for non-phosphorylated neurofilament protein (NPNFP). Scale bar = 1 mm.

### The Pontine Nuclei and the Cerebral Cortex

The pontine nuclei are a critical relay between the cerebral cortex and the cerebellum. The input to the pontine nuclei is from layer 5 pyramidal cells of various regions of the cerebral cortex (Brodal, 1968a,b,c, 1972a,b,c, 1978a,b; Glickstein et al., 1972, 1985; Gibson et al., 1977; Bjaalie, 1986; Legg et al., 1989; Bjaalie and Brodal, 1997; Bjaalie et al., 1997; Brodal and Bjaalie, 1992, 1997; Leergaard and Bjaalie, 2007). The pontine nuclei then project to the cerebellum. The pontine nuclei are implicated in ASD for two reasons, first the documented involvement of their target structure, the cerebellum, and second, the fact that many studies that have found involvement of their input structure, the cerebral cortex. We will therefore briefly review the role of the cerebral cortex in ASD.

### The Cerebellum, the Cerebral Cortex, and ASD

Different studies have found different cortical abnormalities in ASD. One finding has been brain overgrowth in some, but not all, very young ASD children that resolves at later ages (Courchesne et al., 2007, 2011; Libero et al., 2016). Overgrowth of specific regions (especially dorsolateral prefrontal cortex, DLPFC) of the cerebral cortex is a major contributor to differences in overall brain size (Carper and Courchesne, 2005; Courchesne and Pierce, 2005). Projections from the DLPFC to the pontine nuclei have been demonstrated in the monkey (Schmahmann and Pandya, 1997). Other investigators focused on the functional organization of the temporoparietal junction, and suggested differences in connectivity of this region with the cerebellum (Igelstrom et al., 2017). Alterations in cortical circuitry and the structure of cortical columns have also been reported (Casanova et al., 2003). These cortical differences might be reflected in corticopontine projections in the numbers or diameters of axons, or the distribution of projections.

### The Cerebral Cortex and the Corpus Callosum

We have already considered one major efferent pathway from the cerebral cortex, the corticospinal/pontine tract. Another efferent pathway is the corpus callosum (CC). It interconnects the two cerebral hemispheres; neurons of origin are pyramidal cells found primarily in layers 3 and 5 (Jacobson and Trojanowski, 1974). Several studies have noted abnormalities in the CC in ASD, (additional references in Fingher et al., 2017) further evidence that the projections from cortex may be affected.

### The Arcuate Nucleus

Another source of mossy fiber input to the cerebellum is from a structure unique to the human brain, the arcuate nucleus of the medulla (Essick, 1912; Baizer and Broussard, 2010; Baizer, 2014; Baizer et al., 2021). The arcuate has classically been considered a precerebellar structure (Essick, 1912). We have shown its size and shape to be very variable among normal human cases, again a complicating factor in interpreting data from ASD brains. Figure 3 illustrates the variability of the size and shape of the arcuate nucleus in humans. In one report (Bailey et al., 1998) the arcuate was described as “larger than usual” but it was unclear what it was compared to. It too could be added to the list of structures to be analyzed in future postmortem or imaging brain studies.

### Projection Targets of the Cerebellum

Targets of cerebellar outflow include thalamic nuclei and several brainstem structures including the red nucleus, the vestibular nuclear complex (VNC), the IO (Asanuma et al., 1983a,b; Hazrati and Parent, 1992) and the superior colliculus (Roldan and Reinoso-Suarez, 1981). The VNC will be considered in the context of vestibular information processing and the control of eye movements, the IO was discussed above as it is also an input structure.

### Red Nucleus

The red nucleus is a midbrain structure with a role in reaching and grasping (van Kan and McCurdy, 2001, 2002a,b). The red nucleus has two components, a parvocellular and a magnocellular division; the relative sizes of the two components has changed over evolution and the magnocellular component is much smaller in humans than in other mammals (Massion, 1967; Hicks and Onodera, 2012). It has not been specifically mentioned in the neuropathology of ASD, and may not have been examined.

We will now return to the question of possible brainstem involvement in the other functional deficits in autism: control of eye movements, sensory processing of auditory and vestibular information, and control of affect.

### The Brainstem and the Control of Eye Movements

As discussed earlier, abnormal control of eye movements is often mentioned as an ASD symptom. The control of eye movements is mediated by many complexly interconnected brain structures (summary in Figure 4). Key regions include cortical frontal and supplemental eye fields (Robinson and Fuchs, 1969; Schiller et al., 1987; Fukushima et al., 2004; Roesch and Olson, 2005), the flocculus and vermis of the cerebellum (Lisberger and Fuchs, 1974; Kojima et al., 2010a,b,c, 2011), the superior colliculus of the midbrain (Wurtz and Goldberg, 1971, 1972; Stryker and Schiller, 1975), the substantia nigra pars reticulata (SNr; Hikosaka and Wurtz, 1983a,b, 1985) and the four nuclei of the vestibular nuclear complex, the VNC (Chubb and Fuchs, 1982; Chubb et al., 1984; Waespe and Henn, 1977, 1979, 1985). While abnormalities have been described of the cerebellum in ASD, the superior colliculus, the substantia nigra and the VNC have not been implicated in neuropathological studies (Bauman, 1991; Bauman et al., 1995; Bailey et al., 1998; Palmen et al., 2004). There are many other brainstem structures critical in eye movement control include cranial nerve nuclei 3, 4, and 6, (Fuchs and Luschei, 1970, 1971), premotor neurons in midbrain, pons, and medulla (Horn and Büttner-Ennever, 1998), the paramedian pontine reticular formation, (PPRF; Keller, 1974); the nucleus prepositus hypoglossi (PrH; Kaneko, 1992, 1997, 1999); the rostral interstitial nucleus of the medial longitudinal fasciculus (riMLF; Wang and Spencer, 1996; Sparks, 2002) and the Edinger-Westphal nucleus (EW; May et al., 2008). Many of the studies of these nuclei establishing their participation eye movement control have been electrophysiological. It is possible that differences in these structures in ASD brains might be detectable only physiologically, as altered functional circuitry, but not anatomically.

### Vestibular Nuclear Complex

Both functional and anatomical evidence suggest that the vestibular nuclear complex (VNC) may be involved in ASD: (1) The disruptions of vestibular function, (2) The deficits in eye movement control, and (3) Connections with the cerebellum. The eighth cranial nerve distributes vestibular information to the four nuclei of the vestibular nuclear complex (VNC; Figure 5) and to the cerebellum (Barmack, 2003). The nuclei of the VNC are critical for the analysis of vestibular input and are also involved in the generation of vestibular-triggered eye movements like vestibular nystagmus and the vestibulo-ocular reflex, (VOR; Szentagothai, 1950). The VNC also receive projections from the flocculus (Balaban et al., 1981), an eye-movement related part of the cerebellum (Zee et al., 1981). The vestibular nuclei are relatively small structures and could be examined in future neuropathological analysis of ASD brains. We have studied the organization and neurochemical composition of the vestibular nuclear complex in several species including humans, and those data could be used for comparison with VNC organization in ASD brains (Baizer and Broussard, 2010; Baizer et al., 2011a; Baizer, 2014). Figure 6 illustrates neurochemically defined subdivisions in the human VNC.

### The Brainstem and Auditory Processing

There are multiple brainstem nuclei critical for the processing of auditory information including the cochlear nuclei, the nucleus of the trapezoid body, superior olivary complex, nuclei of the lateral lemniscus, and the inferior colliculus (schematic in Figure 7; Pickles, 2015; Smith et al., 2019). There are electrophysiological data (measurement of ABR, auditory brainstem response) suggesting that the brainstem structures may be affected in ASD (Rosenhall et al., 1988; Kwon et al., 2007). An early report found severe brainstem abnormalities in a single case, including aplasia of the superior olivary complex (SOC) and the seventh nerve nucleus (Rodier et al., 1996). Subsequent studies of the medial superior olive (MSO) have found more subtle differences at a cellular level in ASD brains. Quantitative analysis of the MSO showed hypoplasia with fewer, smaller and atypically shaped and oriented neurons (Kulesza and Mangunay, 2008; Smith et al., 2019; Mansour and Kulesza, 2020). Examination of the other components of the SOC also showed abnormalities in neuron numbers and shape (Kulesza et al., 2011; Lukose et al., 2015; Mansour and Kulesza, 2020). Those studies suggest that a similar cellular analysis of the other main brainstem auditory nuclei that interconnect with the SOC (cochlear nuclei, inferior colliculus) might also reveal abnormalities. We have studied the neurochemical organization of the dorsal and ventral cochlear nuclei in humans (Baizer et al., 2014, 2018a); these brain sections are available for comparison with sections from ASD brains. Figures 8A,B shows the dorsal and ventral cochlear nuclei in humans.

### The Brainstem and Neural Substrates of Affect

Several studies have found abnormalities in the amygdala and hippocampus, structures involved in affect and memory (Schumann et al., 2004). However, the regulation of affect also depends on monoaminergic input to the brain. There are two critical brainstem nuclei: the raphe nuclei (cells groups along midline of the brainstem that provide serotonergic input; Hornung, 2003) and the nucleus called the locus coeruleus (in the rostral pons, noradrenergic input; Schwarz and Luo, 2015). Drug therapies for mood disorders in ASD have included SSRIs and SNRIs (selective serotonin or norepinephrine reuptake inhibitors) (Henry et al., 2009; Nanjappa et al., 2020). Amitriptyline, which also affects norepinephrine reuptake, has been used to treat hyperactivity and impulsivity in ASD (Bhatti et al., 2013). One possible interpretation is inadequate production of serotonin and/or norepinephrine in ASD, or abnormalities in the receptors for those transmitters. Several studies provide anatomical and functional evidence for abnormalities in serotonergic function in ASD. Azmitia et al. (2011) found larger numbers of serotonergic fibers in one tract, the medial forebrain bundle, innervating the amygdala, as well as dystrophic (abnormally large diameter) serotonergic fibers in ASD brains. Beversdorf et al. (2012) reported a decrease in serotonin receptor-binding in the thalamus for high-functioning ASD subjects. Wong et al. (2020) showed that drug-manipulated serotonin levels affected levels of limbic system activation of ASD but not control subjects (assessed by fMRI) performing a face-matching task.

### Summary and Future Studies

We have thus identified a large set of brainstem structures that may be implicated in ASD. How might those structures be affected? There could be structural differences, at a macroscopic (size, shape, or organization of a nucleus) or microscopic (differences in cellular characteristics, e.g., neuron size, dendritic tree spread, etc.) level. There could also be functional differences, e.g., in the efficacy of a transmitter, that would be seen at a physiological but not at a structural level. How can we investigate these possibilities? Imaging studies may be able to show abnormalities in the size and shape of larger brain structures, but at present cannot reveal subtle differences in organization or neurochemical changes of smaller ones. Studies of the relatively small brainstem structures are possible through studies of individual brains, optimally from patients whose symptoms and history have been very well-characterized. Ideally, the brains would be from as uniform as possible a population; at minimum details like IQ, and the presence or absence of a seizure disorder would be available. Brains would then be studied by standard histological techniques, including cell, fiber, and immunohistochemical staining.

Immunohistochemistry (IHC) would be essential for studying the serotonergic and noradrenergic transmitter systems. IHC might be useful at looking at aspects of other transmitter systems as well, the density and distribution of receptors or transporters could be examined. Another set of antibodies that might be useful is those to calcium-binding proteins calbindin, calretinin and parvalbumin as their levels may reflect the underlying physiological states of neurons (Blumcke et al., 1990; Celio, 1990; Arai et al., 1991; Baimbridge et al., 1992; Conde et al., 1994; Baurle et al., 1997). The level of the analysis of ASD brains could range from simple examination of cell, fiber, or immunostained sections at the light microscopic level to more quantitative stereological analysis (numbers or packing density of neurons in a structure; for example see Whitney et al., 2009).

However, the analysis needs to be considered carefully. The basic question is simple: do ASD brain structures differ from those in “normal” brains, but the analysis is far from simple. The critical question is how do we define a “normal” brain? There is much variability among human brains in the location, size, shape, and sometimes neurochemical properties of neurons in different brainstem structures (see the data on the IOpr in Baizer et al., 2011b). There are two approaches to selecting comparison data. One is to attempt to obtain and process brains in the same laboratories using the same techniques from controls that are matched for gender, age, IQ, presence of a seizure disorder, etc. The second approach is to compare ASD brain sections with images of “normal” brains as shown either in atlases (Olszewski and Baxter, 1982; Paxinos and Huang, 1995) or in publications (for example Baizer and Broussard, 2010; Baizer et al., 2011b).

It also may be useful to narrow the study population by using a subset of ASD symptoms, e.g., studying only those with particular motor symptoms or eye movement deficits. The problem then is acquiring enough brains to get meaningful results. Another approach is to study a biologically defined population. One example is Fragile X (FXS) syndrome (Garber et al., 2008; Berry-Kravis et al., 2018) which has been used as an animal model of ASD (for example He et al., 2017). However, such studies, in humans or animals, have major limitations in contributing to an understanding of the neuroanatomy of ASD. First, the symptoms of FXS can vary widely, not everyone with FXS is diagnosed as ASD (Garber et al., 2008; Hagerman et al., 2017). Second, FXS causes deficits in a single protein (Fragile X mental retardation protein 1, FMRP, Hagerman et al., 2017). This protein does not have a neuroanatomically limited distribution (Hagerman et al., 2017; Telias, 2019). The mutation is likely to cause widespread functional disruption at the circuit level but it is unlikely that there would be localized structural differences in the brainstem, cerebellum or elsewhere in the brain that could be visualized by neuropathological analysis.

## Conclusion

The topic of this review is the possible role of the brainstem in autism. We have summarized literature on the functions affected in ASD and the brain structures and circuits that mediate those functions. Many of these circuits have brainstem components, and we suggest candidate brainstem nuclei and tracts that may be functionally altered in ASD. However, because of the heterogeneity of possible causes and symptoms of ASD, proving the involvement of these structures may be a very difficult task. It may be that the concept of an autism “spectrum” is useful clinically but misleading in trying to understand the biological basis of ASD. The ASD “spectrum” may not in fact reflect a continuum but instead consist of many separate and independent biological disorders with overlapping manifestations at the behavioral level but diverse neuroanatomic and genetic underpinnings. Progress in genetic analysis is likely to clarify the biological understanding of ASD.

## Data Availability Statement

The raw data supporting the conclusions of this article will be made available by the authors, without undue reservation.

## Ethics Statement

The studies involving human participants were reviewed and approved by the Hamilton Integrated Research Ethics Board (HiREB), McMaster University. The patients/participants provided their written informed consent to participate in this study.

## Author Contributions

JSB read the papers discussed in the literature review, processed the sections shown in the Figures, composed the schematic diagrams, and wrote the manuscript.

## Conflict of Interest

The author declares that the research was conducted in the absence of any commercial or financial relationships that could be construed as a potential conflict of interest.

## Publisher’s Note

All claims expressed in this article are solely those of the authors and do not necessarily represent those of their affiliated organizations, or those of the publisher, the editors and the reviewers. Any product that may be evaluated in this article, or claim that may be made by its manufacturer, is not guaranteed or endorsed by the publisher.
